# Supplementary material for: Patterns of Red and Processed Meat Consumption across North America: A Nationally Representative Cross-Sectional Comparison of Dietary Recalls from Canada, Mexico, and the United States
Source: Int J Environ Res Public Health. 2021 Jan 5;18(1):357. doi: 10.3390/ijerph18010357 (PMC7796493; doi:10.3390/ijerph18010357)
Supplement: Supplementary file 1 [file ijerph-18-00357-s001.pdf]

Supplementary File 2 – Survey Sampling Information

All information below and further details are available from the respective survey user guides [18-20]. Note that for all files, complex survey weights were constructed after data collection to account for missing data and ensure that the survey is nationally- (CCHS, NHANES) or regionally- (ENSANUT) representative.

Canadian Community Health Survey – Nutrition, 2015

Sample Recruitment

"The sample design is multi-stage; each stage will be described in turn. To begin, the 10 provinces were subdivided into a total of 42,340 geographic areas called clusters. These clusters were classified as being either urban or rural in composition. Within each province, urban and rural clusters were selected using systematic probability proportional to size (PPS-SYS) sampling, where the size of each cluster was the number of dwellings it contained. As a result, 1922 clusters were selected.

Within each selected cluster, we created a list frame of all dwellings using the Household Survey Frame Service. For most dwellings, additional socio-economic information was available so we could stratify the dwellings according to the ages of the occupants into seven groups: Between 1 and 4 dwellings were selected in each stratum, resulting in 37,694 dwellings selected across all of the provinces.

A roster of household members within each sampled dwellings was made. One person was then selected using probability proportional to size (PPS) sampling where the size measure was determined prior to collection by methodology to meet sample size objectives by dietary reference intake group (DRI). The PPS size measure initial values were in place for collection occurring between January and June, they were changed for the July-August collection period, and once more for the September to December collection period to optimize the number of respondents obtained in each DRI based on how collection was going up to that point."

Missing Data and Sample Weights

"Dietary recall records were identified as being invalid: 1) due to technical problems when capturing the amounts of food; 2) due to missing meal information; and 3) when a respondent did not know enough detailed information about the food consumed…Several cases [meeting one or more of these criteria] were identified during processing. Because these records were known before the final sample weighting, it was decided that these records would be dropped from files as non-respondents and the sample weights adjusted. Every remaining record on the file has a valid health component and 24-hour dietary recall.

In order for estimates produced from survey data to be representative of the covered population, and not just the sample itself, users must incorporate the survey weights in their calculations. A survey weight is given to each person included in the final sample, that is, the sample of persons having answered the survey. This weight corresponds to the number of persons in the entire population that are represented by the respondent. Several steps are part of the weighting strategy. The following sections describe the weighting process for the survey, including the calculation of the initial weights, the treatment of non-response and the poststratification, where the weights are adjusted to match known population totals. Diagram A presents an overview of the different adjustments that are part of the weighting strategy. A numbering system is used to identify each adjustment and will be used throughout the section. Adjustments applied to household weights are enumerated from H1 to H3, while adjustments applied to person weights are enumerated from P1 to P5."

Diagram A Weighting Strategy Overview

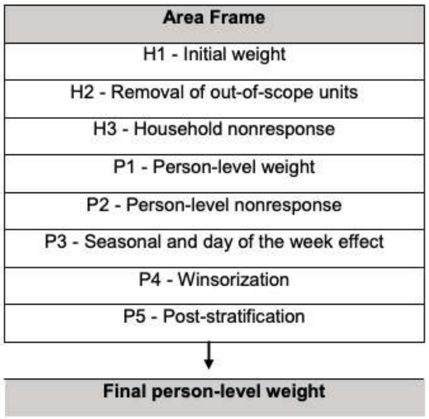

Mexico National Health and Nutrition Survey (English Translation), 2016

Sample Recruitment

" 1. Selection of primary sampling units

The sampling procedure is probabilistic, a characteristic that guarantees the validity of statistical inferences. The sampling stage in the first stage was a list of basic geo-statistical areas (AGEB) built by the National Institute of Geography and Statistics (INEGI). In the urban localities the AGEB list from the 2010 INEGI Census was used; in rural locations the list of rural AGEBs of 2005 with updated information from the 2010 INEGI Census was used. AGEBs were stratified according to two criteria: urbanicity and marginalization. The first stratification criterion was urbanicity category: rural, metropolitan or urban complement. The rural localities are those localities with less than 2,500 inhabitants in the 2010 Census.

The second stratification criterion was defined based on the construction of a social lag index of the AGEB; the index was assigned to each AGEB with a procedure similar to that used by Coneval; The index served to define three groups of AGEB at the national level with different levels (high, medium, low) and containing the same population (33%). Stratification by this index was used to overrepresent AGEBs with greater social deprivations as follows: the high lag group, which represents 33% of the population, was assigned 40% of the sample. In general, oversampling of a stratum increases the design effect due to greater variability of the weights; in this case, the increase was estimated at 3%; Therefore, the estimated Deff = 1.7.

2. Selection of secondary sampling units and dwellings

In urban AGEB, six blocks were selected with probability proportional to their 2010 registered Census population; then in each block six homes were selected via a systematic sampling frame using a housing sketch built by the field team. In the rural AGEB, three locations were selected with probability proportional to their population in the 2010 Census; then, in each rural location, a cluster of 12 dwellings was selected with simple random sampling based on a list of clusters built by the field team.

3. Selection of individuals in households

Whenever possible, from each household in the sample, one member of each population group was selected: adult (20+ years), adolescent (10 to 19 years), school-age (5 to 9 years), and preschool (0 to 4 years). In a second sampling stage, subsamples of the nutrition sampling were defined: venous blood, one 24-hour recall and second 24-hour recall. The 24-hour recall subsample resulted from sampling 30% of preschoolers and schoolchildren, 50% of adolescents, and 30% of adults with venous blood draws. The nutrition subsamples were selected by systematic sampling, for example: the subsample to obtain venous blood consisted of the sequence: {1,3,5,6,7,9,11,13,15,16,17,19 .. .} of the homes

visited by each interviewer. Similar sequences were defined for each nutrition subsample.”

Missing Data and Survey Weights

The response rate for adult participants was 91.7%. Probabilistic complex survey weights accounting for sampling schema and non-response were constructed after survey completion. “Figure 1 compares the unweighted age pyramid with the weighted age pyramid of the INEGI Inter-Census Survey 2015; subsection a suggests an underreporting of the population aged 20 to 39. Subsection b shows that the weighting process brings the weighted age pyramid closer to the unweighted age pyramid of the INEGI Inter-Census Survey 2015.”

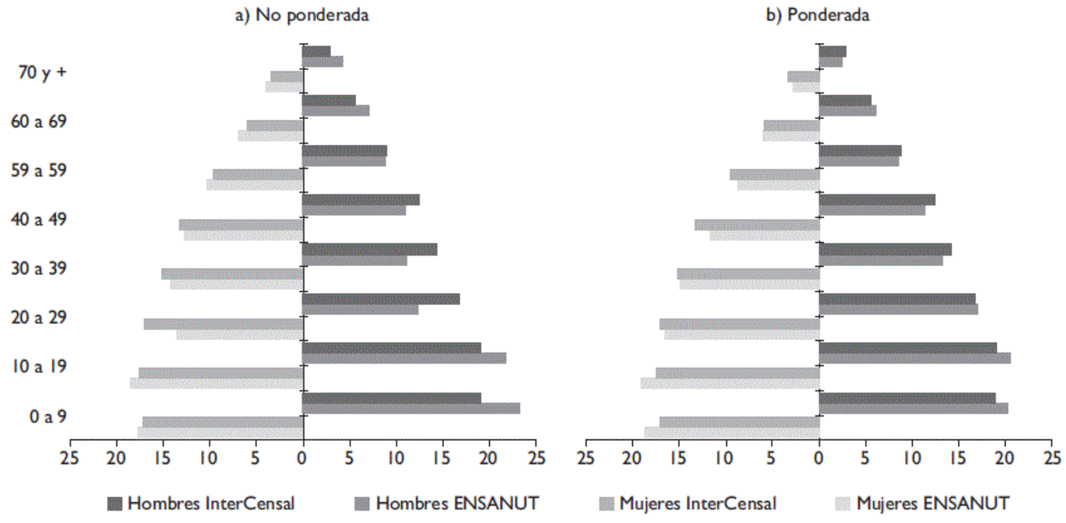

Figura 1. Comparación de la pirámide de edad a) no ponderada y b) ponderada de la Ensanut-MC 2016 con la pirámide ponderada de la Encuesta InterCensal. México, INEGI, 2015

United States National Health and Nutrition Examination Survey, 2013-2014 & 2015-2016

Sample Selection

The NHANES sample represents the noninstitutionalized civilian population residing in the 50 states and the District of Columbia. Since 1999, the sample design has consisted of multi-year, stratified, clustered four-stage samples, with data release in 2-year cycles. The NHANES sample is drawn in four stages: (a) PSUs (counties, groups of tracts within counties, or combinations of adjacent counties), (b) segments within PSUs (census blocks or combinations of blocks), (c) dwelling units (DUs) (households) within segments, and (d) individuals within households. PSUs are sampled from all U.S. counties. Screening is conducted at the DU level to identify sampled persons (SPs), based on oversampling criteria. NHANES 2015–2018 oversampled some subgroups to increase precision for subgroup estimates. The population subgroups chosen for oversampling directly determine the sampling domains used to select the sample at all stages. Specific NHANES sample designs, including specifications for clustering, stratification, and oversampling population subgroups, have changed over time. The set of domains for which specified reliability was desired in NHANES 2015–2018 consisted of sex-age groups for Hispanic persons; non-Hispanic black persons; and non-Hispanic, non-black Asian (referred to as non-Hispanic Asian) persons, and income-sex-age groups for the

remainder of the U.S. population. Specifically, the oversampled subgroups in the 2015–2018 survey were:

- Hispanic persons;
- Non-Hispanic black persons;
- Non-Hispanic, non-black Asian persons;
- Non-Hispanic white persons and persons of other races and ethnicities at or below 130% of the federal poverty level (2013-2014 cycle) or 185% of the federal poverty level (2015-2016 cycle); and
- Non-Hispanic white persons and persons of other races and ethnicities aged 0–11 years or 80 years and over.

Missing Data and Sample Weights

Weighting of the NHANES data produces estimates representative of the civilian resident noninstitutionalized U.S. population. The weighting of sample data permits analysts to produce estimates of the statistics that would have been obtained if the entire eligible population had been surveyed. Sample weights can be considered measures of the number of persons in the target population represented by the particular participant. Weighting takes into account several features of the survey: the differential probabilities of selection for the sampling domains, survey nonresponse, and differences between the final sample distribution and the target population distribution. Each of the three levels of data collection for NHANES (screening, interview, examination) has a response rate. As a result, sample weights are calculated for each level of data collection.

The NHANES sample weighting is carried out in three steps. The first step involves the computation of stage base weights to compensate for unequal probabilities of selection for the sampling domains. The second step adjusts for nonresponse to reduce the potential bias. In the third step, the sample weights are calibrated to the reference population. Calibration is used to compensate for possible coverage differences from the eligible population and to reduce variances in the estimation procedure. The nonresponse and calibration steps are performed at each level of data collection: the screening, interview, and examination. The new weight for each step in the adjustment is the product of the weight before the adjustment and the adjustment factor of the step.

Adjustment factors are calculated within adjustment cells defined by characteristics available for both sample participants and nonrespondents at that step. Because available information differs for each step, adjustment cells differ for each step."
